# Health-related quality of life, lifestyle habits and chronic pain in individuals with knee pain – a 2-year follow-up study

**DOI:** 10.1080/02813432.2025.2452916

**Published:** 2025-01-20

**Authors:** Charlotte Sylwander, Emma Haglund, Ingrid Larsson, Maria L. E. Andersson

**Affiliations:** a School of Health and Welfare, Halmstad University, Halmstad, Sweden; b Spenshult Research and Development Centre, Halmstad, Sweden; c Department of Clinical Sciences, Section of Rheumatology, Lund University, Lund, Sweden; d Department of Environmental and Biosciences School of Business, Innovation and Sustainability, Halmstad University, Halmstad, Sweden

**Keywords:** Knee pain, chronic pain, health-related quality of life, lifestyle habits, overweight, prevention

## Abstract

**Introduction:**

Knee pain increases the risk of developing chronic widespread pain (CWP) and knee osteoarthritis (KOA). The prevalence of CWP and KOA has increased, and there is a need for early prevention. Therefore, the aim was to examine the associations of health-related quality of life (HRQoL) and lifestyle habits with chronic pain at a two-year follow-up in individuals with knee pain.

**Methods:**

A two-year longitudinal cohort study including 251 individuals aged 30–60 years reporting knee pain at baseline. HRQoL was measured via the Short-Form General Health Survey (SF-36), and lifestyle habits included questions on overweight, physical activity, diet, alcohol and tobacco use. Pain was assessed with a pain mannequin. Differences in health status and lifestyle habits over time in groups with unchanged no chronic pain (NCP), transitioned to less and more pain, and unchanged CWP were analysed using Wilcoxon’s, McNemar’s and Friedman’s tests. Multinominal regression analysis was performed to study associations with reporting chronic pain at follow-up.

**Results:**

Reporting better HRQoL across various SF-36 concepts and normal weight at baseline was associated with reporting NCP after two years. A few changes were made regarding HRQoL and lifestyle habits over the course of two years, but an increase in general health was associated with transitioning to less pain.

**Conclusions:**

During primary care visits for knee pain with a combination of overweight or lower HRQoL, individuals should receive comprehensive attention to prevent the development of CWP. Future studies should investigate the associations further.

## Introduction

Around 30% of the adult population worldwide suffers from chronic musculoskeletal pain and it is one of the most common reasons to seek health-care [1]. The more severe condition, chronic widespread pain (CWP), affects up to 15% of the general population [2], but among individuals with knee pain, the prevalence is 30% [3]. In addition to an increased risk of developing CWP, a majority of individuals with knee pain develop radiographic changes in the knee over time (regardless of CWP) [4]. Knee osteoarthritis (KOA) (clinical or radiographic) is a common disease in the general population, causing pain and disability [5].

CWP or KOA can deeply affect the individual, causing reduced health-related quality of life (HRQoL), impairments, activity limitations and participation restrictions with a high proportion of sick leave [6,7]. But not everyone has pain-related interference, defined as ‘perceived disruption in daily activities, relationships, roles, and employment resulting from pain’ [8], which is highlighted in the global call for more health-promotive research in chronic pain [9]. The call urges scientists to focus on increasing the understanding of how HRQoL and lifestyle habits impact individuals at risk of developing chronic pain.

Adopting healthy lifestyle habits, such as engaging in physical activity and achieving weight loss when overweight, can reduce the risk of chronic pain and improve HRQoL when suffering from chronic pain [10,11]. These lifestyle changes are also among the recommended self-management strategies for chronic pain and KOA [1,12], and adhering to them can lead to reduced pain, improved physical function (PF) and enhanced HRQoL [13,14]. Previous research reports that the prevalence of chronic pain is 20% higher among overweight individuals compared to normal-weight individuals, and the prevalence increases with obesity [15]. Increased body mass is also a risk factor for developing KOA and is associated with the progression and severity of osteoarthritis [15].

Research predicts a rise in chronic pain prevalence with an ageing population and reduced physical activity [16], and the prevalence of KOA has increased in recent years [17]. A prior study on the cohort used in this paper found that one in seven individuals with clinical KOA progressed to radiographic KOA within two years [18]. In addition, worse pain symptoms, overweight and HRQoL were associated with the progression. Another study found that several healthy lifestyle habits could be protective for the development of chronic low back and neck pain in a four-year perspective in the general population [10].

Considering the expected increasing prevalence of chronic pain and KOA, a proactive approach to prevention is essential. As of today, treatment for chronic pain/CWP often begins late in the disease process, and earlier treatment is emphasised [19]. The same occurs for KOA, missing the potential window for a better outcome [20]. It is well-known that HRQoL and lifestyle habits are associated with established KOA and chronic pain, but less is known of how much and how fast they impact a possible progression or degression. Hence, studies examining earlier stages of the conditions are needed to gain knowledge of the impact HRQoL, and lifestyle habits may have over different periods of time. Individuals with knee pain are considered suitable to study due to the increased risk of developing CWP and KOA. In the current study, we focus on chronic pain and the aim was to examine the associations of HRQoL and lifestyle habits with chronic pain at a two-year follow-up in individuals with knee pain.

## Materials and methods

### Study design and participants

The study is part of the Halland Osteoarthritis Cohort study (HALLOA) following individuals with knee pain over five years [21]. The current study is one of several sub-studies targeting the overall aim ‘to study pain development and pain pressure thresholds in relation to lifestyle, depression and health-related quality of life in individuals with KOA’ [18]. Baseline data were collected during 2017–2020, and the two-year follow-up (2–3 years after baseline) during 2019–2022. Participants were recruited foremost via local newspaper ads, but also through primary health care clinics. Inclusion criteria were current knee pain, age 30–60 years, no prior diagnosis of KOA, and no rheumatologic disorders or cruciate ligament injuries. In total, 306 individuals were included, and at the two-year follow-up, 251 individuals participated (70% women, mean age 52 ± 8.5 years). The dropout rate was 18%. The non-responders were mostly women (82%), and more had a high visceral fat area (VFA >100 cm^2^) compared to the included individuals (*p* = .038). No other difference was found (*p* > .05).

### Outcome measures

#### Health-related quality of life assessment

HRQoL was assessed by the Swedish version of the Short-Form General Health Survey (SF-36) questionnaires [22]. The SF-36 questionnaire has eight different health concepts: PF, role function – physical aspect (RP), bodily pain (BP), general health perception (GH), vitality (VT), social functioning (SF), role function – emotion aspect (RE) and mental health (MH). Each concept has a score that ranges from 0 to 100, and a higher score indicates a better HRQoL [22].

#### Lifestyle assessments

The lifestyle habits of physical activity, diet, tobacco and alcohol habits were assessed using the Swedish National Board of Health and Welfare’s questions about living habits [23] together with a separate question about sedentary, measured in hours (h). *Physical activity* was measured with two questions, including (1) amount (minutes) physical exercise (vigorous) and (2) non-exercise physical activity (moderate intensity). Questions about *diet* included intake of vegetables, fruits/berries, fish/seafood, sweets/snacks/pastries/soda and breakfast habits (number of days per week). *Tobacco* was measured with one question about smoking habits (never smoked, former or current smoker) and one about snuffing (never used snuff, former or current snuff user). *Alcohol intake* was measured with two questions: (1) how many units of alcohol per week and (2) how often you drink four units or more per week.

Overweight, a lifestyle-related factor, was assessed by body mass index (BMI, kg/m^2^), VFA (cm^2^) and waist circumstance (cm). BMI and VFA were measured using a multifrequency bioelectrical impedance analysis (InBody 770^®^, Seoul, South Korea). Central obesity measured by waist circumstance was classified as ≥80 cm in women and ≥94 cm in men, according to the International Diabetes Federation [24].

#### Pain assessments

Pain distribution was assessed with a pain mannequin according to the 2019 criteria for CWP [25]. According to the criteria, CWP is defined as having pain for ≥3 months in at least four of five body regions (right and left upper and lower body, and axial) and at least seven painful sites out of 15. If the criteria for CWP are not met, the pain is referred to as chronic regional pain (CRP) if having pain for ≥3 months. At baseline, all individuals had knee pain, but in the 2019 criteria for CWP, the knee regions were excluded. In this study, the knees continued to be excluded to detect pain other than knee pain. Based on the answers in the pain mannequin, the individuals were divided into three groups: no chronic pain (NCP) other than knee pain, CRP and CWP.

#### Knee osteoarthritis

Radiographic KOA was assessed with X-ray and defined according to the Ahlbäck five grading scale [26]. A result of ≥1 was considered radiographic KOA.

### Statistical analysis

Baseline characteristics were socio-demographics, HRQoL, lifestyle habits and rKOA. The obesity variables showed no normal distribution, whereas nonparametric statistics were used. The results were presented as median with interquartile range (IQR). A Chi-square test and the Mann–Whitney *U*-test were used to analyse differences between groups. The paired analyses of Wilcoxon’s, McNemar’s and Friedman’s ANOVA tests were used to study change over time regarding HRQoL and lifestyle variables in four groups. The groups were formed based on the reported pain group (NCP, CRP and CWP) at baseline and follow-up. They consisted of (1) unchanged NCP, (2) reporting less pain at follow-up compared to baseline, (3) reporting more pain at follow-up and (4) unchanged CWP.

Associations to reporting CRP or CWP were analysed using univariate multinominal logistic regressions, with NCP as a reference and contrary associations to reporting NCP or CRP with CWP as a reference. Variables with a *p* value of ≤.25 were included in the multivariate analysis adjusted for age and sex [27]. The lifestyle variables central obesity, physical activity, diet, tobacco use and alcohol intake were dichotomised as healthy or unhealthy in the regression analysis. A healthy level of physical activity meant meeting the WHO recommendations of 150–300 min of moderate-intensity and/or 75–150 min of vigorous activity (MVPA). A healthy diet was defined as eating vegetables and fruit daily, fish twice a week, breakfast most days and pastries a few times per week. A healthy alcohol intake was ≤4 units per week, and healthy tobacco use (smoking and snuff) meant no smoking or snuff use. Due to the low prevalence of smoking and snuff use, i.e. lack of power, these were not included in the regression analysis. Based on the sub-scale GH in SF-36, there is at least 80% power for comparisons between all three pain groups. Minimal clinically relevant difference in SF-36 was considered a ±10 change in score [28]. Results were considered significant if *p* ≤ .05. All analyses were performed in the IBM SPSS 24 statistical package for Windows (released 2016; IBM Corp., Armonk, NY).

### Ethical considerations

The study adhered to the Helsinki Declaration and was approved by the Swedish Ethics Review Authority (Dnr 2016/816; 2017/205). All participants signed a written informed consent document.

## Results

At baseline, almost half of the individuals with knee pain (48%) had higher education levels, with no difference between the three pain groups (Table 1). Most were native-born in Sweden (87%), and 18.5% lived alone. No significant differences between the pain groups were found. Individuals reporting chronic pain (CRP or CWP) had worse HRQoL in most of the SF-36 sub-scales, and the group with CWP reported the lowest health scores compared to those with NCP (*p* value between .016 and ≤.001).

**Table 1.** Baseline characteristics for the whole sample and the three pain groups, presented as median and interquartile range (IQR) or as *n* (%).

	Total, *n* (%) *n* = 251	Pain group			*p* Value	Pairwise comparisons
		NCP *n* = 52	CRP *n* = 153	CWP *n* = 33		
Age, mean years (SD)	52 (8.5)	52 (8.5)	52 (8)	52 (8)	.918	
Women, *n* (%)	168 (67)	29 (56)	102 (67)	27 (82)	.046	NCP-CRP (*p* = .159); NCP-CWP (*p* < .014); CRP-CWP (*p* = .088)
Education, *n* (%)					.960	
Compulsory school	29 (12)	5 (10)	19 (13)	5 (15)		
Secondary	95 (40)	21 (40)	61 (40)	13 (39)		
University	113 (48)	26 (50)	72 (47)	15 (46)		
Native-born, *n* (%)	206 (87)	48 (92)	131 (86)	27 (82)	.328	
Living alone, *n* (%)	44 (18.5)	7 (13.5)	30 (20)	7 (21)	.559	
HRQoL (SF36), median (IQR)						
PF	85 (20)	90 (20)	85 (20)	65 (25)	<.001	NCP-CRP (*p* = .018); NCP-CWP (*p* < .001); CRP-CWP (*p* = .006)
RP	100 (50)	100 (0)	100 (50)	50 (75)	<.001	NCP-CRP (*p* = .006); NCP-CWP (*p* < .001); CRP-CWP (*p* < .001)
BP	62 (33)	72 (22)	62 (31)	41 (28)	<.001	NCP-CRP (*p* < .001); NCP-CWP (*p* < .001); CRP-CWP (*p* < .001)
GH	72 (30)	82 (15)	72 (28)	54 (25)	<.001	NCP-CRP (*p* = .001); NCP-CWP (*p* < .001); CRP-CWP (*p* < .001)
VT	60 (40)	75 (25)	60 (30)	43 (20)	<.001	NCP-CRP (*p* = .003); NCP-CWP (*p* < .001); CRP-CWP (*p* = .001)
SF	100 (25)	100 (0)	100 (25)	75 (47)	<.001	NCP-CRP (*p* = .043); NCP-CWP (*p* < .001); CRP-CWP (*p* < .001)
RE	100 (33)	100 (0)	100 (33)	67 (92)	.002	NCP-CRP (*p* = .221); NCP-CWP (*p* = .001); CRP-CWP (*p* = .029)
MH	84 (24)	92 (16)	84 (23)	71 (34)	<.001	NCP-CRP (*p* = .008); NCP-CWP (*p* < .001); CRP-CWP (*p* = .067)
BMI^a^, *n* (%)					.027	NCP-CRP (*p* = .274); NCP-CWP (*p* < .009); CRP-CWP (*p* = .023)
Normal	110 (45)	27 (54)	68 (45)	10 (31)		
Overweight	85 (34.5)	16 (32)	56 (37)	9 (28)		
Obese	51 (20.5)	7 (14)	27 (18)	13 (41)		
VFA, cm^2^, median (IQR)	99 (69)	76 (59)	98 (64)	146 (104)	<.001	NCP-CRP (*p* = .035); NCP-CWP (*p* < .001); CRP-CWP (*p* = .017)
Central obesity^b^, *n* (%)	197 (80)	34 (69)	125 (83)	28 (85)	.095	
MVPA^c^, *n* (%)	170 (71)	37 (71)	112 (73)	21 (64)	.543	
Healthy diet^d^, *n* (%)	58 (24)	15 (29)	36 (23.5)	7 (21)	.648	
Smoker, *n* (%)	19 (8)	4 (8)	11 (7)	4 (12)	.579	
Snuff user, *n* (%)	12 (5)	3 (6)	8 (5)	1 (3)	.834	
Alcohol intake, *n* (%)					.513	
<1 unit/week	71 (34)	16 (31)	50 (33)	15 (46)		
1–4 units/week	120 (51)	30 (58)	77 (51)	13 (39)		
≥5 units/week	35 (15)	6 (11)	24 (16)	5 (15)		
Radiographic KOA^e^, *n* (%)	57 (23)	11 (21)	33 (22)	10 (30)	.555	

SD: standard deviation; NCP: no chronic pain; CRP: chronic regional pain; CWP: chronic widespread pain; HRQoL: health-related quality of life; PF: physical functioning; RP: role function – physical aspect; BP: bodily pain; GH: general health perception; VT: vitality; SF: social functioning; RE: role function – emotion aspect; MH: mental health; BMI: body mass index; VFA: visceral fat area; MVPA: being moderate and/or vigorous physical active; KOA: knee osteoarthritis.

Analysed with Chi-square test, Mann–Whitney’s *U*-test and Kruskal–Wallis test with pairwise comparisons (pain groups at baseline, missing *n* = 13).

^a^ Normal BMI = 18.5–24.9 kg/m^2^; overweight = 25.0–29.9; obesity ≥30.0.

^b^ Central obesity = classified in accordance with IDF as waist circumference ≥94 cm in men and ≥80 cm in women.

^c^ WHO recommendations: 150–300 min of moderate-intensity and/or 75–150 min of vigorous-intensity.

^d^ Vegetables and fruit every day, fish 2/week, breakfast most days, pastries a few times/week.

^e^ Having a score ≥1 on the Ahlbäck scale for knee osteoarthritis.

The presence of overweight and obesity was high. According to BMI, 34.5% were overweight and 20.5% were obese in the whole sample, and 41% were obese in the CWP group (*p* = .027). The group’s median VFA was 99 (IQR 69) cm^2^, and according to waist circumference, 80% of the sample had central obesity. Around 70% met the WHO recommendations for physical activity, which was higher among those with normal weight than those with obesity (78% vs. 57%, *p* = .032). Only 24% reported having a healthy diet, and the presence of a healthy diet was overall low in all pain groups. The prevalence of smokers and snuff users was overall low, and around 15% reported drinking five or more units per week.

**Figure 1.**
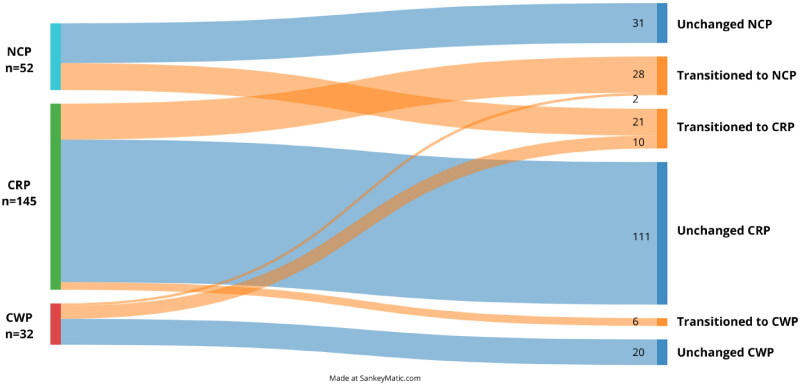
Pain groups and transitions from baseline to the two-year follow-up (*n* = 229, missing = 22).

Few pain transitions were made between the baseline and the two-year follow-up, and most remained in the same pain group (Figure 1). In total, 229 individuals completed the pain mannequin at baseline and the two-year follow-up (22 missing). All reported knee pain at baseline, and the pain distribution represents pain other than the knees. After two years, 13% transitioned to less pain and 9% transitioned to more pain. Among those reporting CWP at baseline, 37% transitioned to less pain at the follow-up, 31% CRP and 6% NCP. Within the group reporting CRP at baseline, 19% transitioned NCP. At the two-year follow-up, 4% had transitioned to CWP, previously reporting CRP, and 40% to CRP, previously reporting NCP.

Overall, few changes in HRQoL and lifestyle habits were found between baseline and the two-year follow-up among individuals who reported unchanged NCP, transitioned to less pain or more pain, and unchanged CWP (Table 2). The group that transitioned to less pain at the follow-up reported better GH after two years compared to baseline (*p* = .001). On the contrary, they scored worse in BP, but there was not a clinically relevant change in score (*p* = .045). The group with unchanged CWP scored better in RP (*p* = .014) and VT (*p* = .016).

**Table 2.** Overview of HRQoL and lifestyle habits over time from baseline to the two-year follow-up in different pain groups (unchanged pain and transitioning to more or less pain).

	Unchanged NCP *n* = 31				Transition to less pain *n* = 40				Transition to more pain *n* = 27				Unchanged CWP *n* = 20			
	*n*	Baseline	2-year	*p* Value	*n*	Baseline	2-year	*p* Value	*n*	Baseline	2-year	*p* Value	*n*	Baseline	2-year	*p* Value
Women, *n* (%)	31	17 (55)			40	26 (65)			27	16 (59)			20	16 (80)		
HRQoL (SF-36), median (IQR)					38				25				18			
PF		90 (10)	90 (5)	.312		85 (21)	84 (26)	.917		85 (23)	85 (30)	.259		65 (26)	70 (25)	.344
RP		100 (0)	100 (0)	.942		100 (31)	100 (25)	.697		100 (25)	100 (38)	.499		13 (75)	50 (63)	.014
BP		73 (33)	74 (12)	.132		62 (22)	67 (33)	.045		62 (33)	62 (38)	.150		31 (22)	41 (15)	.161
GH		82 (15)	87 (23)	.553		72 (26)	82 (26)	.001		82 (25)	77 (35)	.192		50 (27)	51 (30)	.492
VT		75 (21)	75 (20)	.465		58 (30)	65 (30)	.128		65 (43)	80 (45)	.419		35 (38)	48 (38)	.016
SF		100 (0)	100 (0)	.603		100 (25)	100 (28)	.955		100 (19)	100 (19)	.391		69 (63)	81 (50)	.283
RE		100 (8)	100 (0)	.527		100 (33)	100 (33)	.674		100 (0)	100 (17)	.279		83 (100)	100 (75)	.303
MH		92 (12)	92 (9)	.548		80 (17)	84 (24)	.993		80 (24)	88 (18)	.355		69 (41)	78 (32)	.099
BMI^a^, *n* (%)	30			.317	38			.414	26			.655	20			.317
Normal		17 (56.7)	17 (57)			15 (39)	16 (42)			12 (46)	12 (46)			5 (25)	6 (30)	
Overweight		8 (26.7)	9 (30)			14 (37)	10 (26)			10 (39)	11 (42)			6 (30)	6 (30)	
Obese		5 (16.7)	4 (13)			9 (24)	12 (32)			4 (15)	3 (12)			9 (45)	8 (40)	
VFA, cm^2^, median (IQR)	31	70 (61)	75 (54)	.299	38	111 (70)	118 (74)	.094	25	90 (65)	95 (68)	.367	18	162 (113)	173 (118)	.478
Central obesity^b^, *n* (%)	30	21 (70)	24 (80)	.125	39	33 (85)	33 (85)	1.000	24	16 (67)	16 (67)	1.000	19	16 (84)	17 (90)	1.000
MVPA^c^, *n* (%)	31	22 (71)	23 (74)	.014	40	28 (70)	23 (58)	.063	27	19 (70)	18 (67)	.041	20	14 (70)	13 (65)	.118
Healthy diet^d^, *n* (%)	31	7 (24)	5 (16)	.625	40	4 (10)	4 (10)	1.000	27	10 (37)	6 (22)	.289	20	6 (30)	7 (35)	1.000
Alcohol intake, *n* (%)	31			.414	38			.317	27			1.000	19			.655
<1 unit/week		10 (32)	10 (32)			13 (34)	13 (34)			6 (22)	9 (33)			10 (53)	10 (53)	
1–4 units/week		15 (48.5)	17 (55)			17 (45)	19 (50)			21 (78)	15 (56)			7 (37)	7 (37)	
≥5 units/week		6 (19.5)	4 (13)			8 (21)	6 (16)			0	3 (11)			2 (10)	2 (10)	
KOA^e^, *n* (%)	30	5 (17)	9 (30)	.125	36	15 (39)	17 (44)	.500	25	8 (32)	9 (36)	1.000	19	6 (32)	7 (37)	1.000

NCP: no chronic pain; CRP: chronic regional pain; CWP: chronic widespread pain; HRQoL: health-related quality of life; PF: physical functioning; RP: role function – physical aspect; BP: bodily pain; GH: general health perception; VT: vitality; SF: social functioning; RE: role function – emotion aspect; MH: mental health; BMI: body mass index; VFA: visceral fat area; MVPA: being moderate and/or vigorous physical active; KOA: radiographic knee osteoarthritis.

Presented as median and interquartile range (IQR) or as numbers, *n* (%). Analysed with Wilcoxon’s, McNemar’s and Freidman’s ANOVA tests.

^a^ Normal BMI = 18.5–24.9 kg/m^2^; overweight = 25.0–29.9; obesity ≥30.0.

^b^ Central obesity = classified in accordance with IDF as waist circumference ≥94 cm in men and ≥80 cm in women.

^c^ WHO recommendations: 150–300 min of moderate-intensity and/or 75–150 min of vigorous-intensity.

^d^ Vegetables and fruit every day, fish 2/week, breakfast most days, pastries a few times/week.

^e^ Having a score ≥1 on the Ahlbäck scale for KOA.

The groups with unchanged NCP and transition to more pain showed a statistically significant change in numbers meeting the recommendations for physical activity.

HRQoL and lifestyle habits at baseline that were associated with reporting CRP or CWP, in addition to knee pain, at the two-year follow-up are presented in Table 3. Reporting lower PF, limited RP, more pain (BP), worse GH and lower SF at baseline were associated with reporting CRP two years after. No association with any of the lifestyle habits was found. Reporting a lower score in all eight SF-36 sub-scales at baseline was associated with CWP at the two-year follow-up. Additionally, higher BMI and VFA at baseline were associated with reporting CWP. The results remained when adjusting for age and sex (Table 3).

**Table 3.** Associations with sociodemographic, HRQoL and lifestyle habits at inclusion and reporting CRP and CWP in addition to knee pain at the two-year follow-up.

	Univariate multinominal logistic regression								Adjusted for age and sex							
	CRP				CWP				CRP				CWP			
	*n*	OR	95% CI	*p* Value	*n*	OR	95% CI	*p* Value	*n*	OR	95% CI	*p* Value	*n*	OR	95% CI	*p* Value
Age	146	1.033	0.996–1.071	.078	27	1.035	0.977–1.097	.239								
Sex, female	146	1.625	0.883–2.992	.119	27	2.700	0.957–7.615	.060								
Education	142				25				142				25			
University		1				1				1				1		
Secondary		0.753	0.400–1.415	.377		1.062	0.390–2.891	.906		0.792	0.403–1.555	.498		1.259	0.435–3.642	.671
Compulsory		2.162	0.680–6.870	.191		2.800	0.587–13.361	.197		2.336	0.679–8.037	.178		3.874	0.709–21.153	.118
Emigrated	142	1.503	0.572–3.949	.409	26	2.183	0.601–7.920	.235					26	2.622	0.698–9.856	.154
Living together	142	0.982	0.450–2.193	.982	26	0.924	0.286–2.988	.895								
HRQoL, SF36	140				26				140				26			
PF		0.958	0.934–0.983	.001		0.929	0.899–0.961	<.001		0.963	0.938–0.988	.004		0.934	0.903–0.966	<.001
RP		0.981	0.969–0.994	.003		0.960	0.946–0.975	<.001		0.991	0.968–0.993	.002		0.959	0.944–0.974	<.001
BP		0.976	0.960–0.993	.005		0.925	0.898–0.952	<.001		0.975	0.958–0.992	.004		0.923	0.896–0.951	<.001
GH		0.993	0.977–1.010	.404		0.947	0.924–0.971	<.001		0.990	0.973–1.007	.235		0.940	0.916–0.966	<.001
VT		0.984	0.970–0.999	.031		0.961	0.940–0.981	<.001		0.980	0.964–0.995	.011		0.943	0.921–0.966	<.001
SF		0.985	0.967–1.003	.107		0.956	0.936–0.977	<.001		0.983	0.964–1.002	.077		0.954	0.933–0.976	<.001
RE		0.994	0.984–1.004	.232		0.981	0.968–0.993	.003		0.991	0.980–1.002	.098		0.977	0.964–0.990	<.001
MH		0.978	0.959–0.998	.030		0.960	0.936–0.985	.002		0.955	0.955–0.995	.015		0.955	0.930–0.981	<.001
BMI^a^	143				27								27			
Normal		1				1								1		
Overweight		1.178	0.599–2.319	.635		1.657	0.518–5.304	.395						2.141	0.634–7.236	.220
Obese		1.113	0.486–2.543	.801		4.519	1.414–14.448	.011						4.954	1.516–16.182	.008
BMI, kg/m^2^	143	1.039	0.968–1.115	.292	27	1.122	1.021–1.233	.017					27	1.133	1.031–1.244	.009
VFA, cm^2^	142	1.004	0.998–1.011	.177	27	1.015	1.006–1.024	.001	142	1.004	0.997–1.010	.249	27	1.013	1.004–1.022	.003
MVPA^b^	142	1			26	1										
Not MVPA		1.263	0.645–2.473	.495		1.250	0.456–3.430	.665								
Healthy diet^c^	142	1			26	1			142	1			26	1		
Unhealthy		0.602	0.284–1.276	.189		0.495	0.172–1.426	.193		0.668	0.310–1.440	.304		0.588	0.200–1.730	.335
Healthy alcohol intake^d^	142	1			26	1										
Unhealthy		0.880	0.462–1.676	.696		0.741	0.284–1.935	.541								
No KOA^e^	144	1.351	0.672–2.715	.398	26	0.800	0.292–2.195	.665								

NCP: no chronic pain; CRP: chronic regional pain; CWP: chronic widespread pain; HRQoL: health-related quality of life; PF: physical functioning; RP: role function – physical aspect; BP: bodily pain; GH: general health perception; VT: vitality; SF: social functioning; RE: role function – emotion aspect; MH: mental health; BMI: body mass index; VFA: visceral fat area; MVPA: being moderate and/or vigorous physical active; KOA: radiographic knee osteoarthritis.

Analysed by multinominal logistic regression (NCP as reference. *n* = 61). Variables with a *p* value of ≤.25 were included in the multivariate analysis adjusted for age and sex.

^a^ Normal BMI = 18.5–24.9 kg/m^2^; overweight = 25.0–29.9; obesity ≥30.0.

^b^ WHO recommendations: 150–300 min of moderate-intensity and/or 75–150 min of vigorous-intensity.

^c^ Vegetables and fruit every day, fish 2/week, breakfast most days, pastries a few times/week.

^d^ Healthy intake = ≤4 units/week.

^e^ Having a score <1 on the Ahlbäck scale for knee osteoarthritis.

Similar results were found when analysing associations to reporting NCP and CRP at the two-year follow-up, with CWP as reference (Table 4). Reporting NCP was associated with reporting a better score in all eight SF-36 sub-scales at baseline than CRP and CWP. The same result followed for reporting CRP, except MH was not associated. At the two-year follow-up, reporting a normal weight, including lower BMI and VFA at baseline, was associated with NCP and CRP. The results remained when adjusting for age and sex (Table 4).

**Table 4.** Associations with sociodemographic, HRQoL and lifestyle habits at inclusion and reporting NCP and CRP in addition to knee pain at the two-year follow-up.

	Univariate multinominal logistic regression								Adjusted for age and sex							
	NCP				CRP				NCP				CRP			
	*n*	OR	95% CI	*p* Value	*n*	OR	95% CI	*p* Value	*n*	OR	95% CI	*p* Value	*n*	OR	95% CI	*p* Value
Age	62	0.966	0.912–1.023	.239	146	0.998	0.946–1.053	.941								
Sex, female	62	0.370	0.131–1.045	.060	146	0.306	0.228–1.590	.306								
Education	61				142				61							
Compulsory		1				1				1						
Secondary		2.636	0.560–12.421	.197		0.918	0.263–3.20	.687		3.077	0.623–15.195	.118				
University		2.800	0.587–13.361	.220		1.295	60.368–4.560	.893		3.874	0.709–21.153	.168				
Emigrated	61	0.485	0.126–1.663	.235	142	0.689	0.233–2.035	.500	61	0.381	0.101–1.433	.154				
Living together	61	1.082	0.335–3.499	.895	142	1.062	0.367–3.499	.895								
HRQoL, SF36	61				140				61				140			
PF		1.076	1.041–1.112	<.001		1.031	1.006–1.05	.014		1.071	1.035–1.107	<.001		1.031	1.005–1.057	.018
RP		1.041	1.025–1.057	<.001		1.022	71.011–1.03	<.001		1.043	1.026–1.059	<.001		1.022	1.011–1.034	<.001
BP		1.082	1.082–1.113	<.001		1.056	31.029–1.08	<.001		1.083	1.052–1.115	<.001		1.056	1.029–1.083	<.001
GH		1.055	1.029–1.082	<.001		1.048	31.025–1.07	<.001		1.063	1.035–1.092	<.001		1.053	1.028–1.078	<.001
VT		1.053	1.029–1.077	<.001		1.036	11.016–1.05	<.001		1.060	1.035–1.086	<.001		1.038	1.017–1.060	<.001
SF		1.046	1.023–1.069	<.001		1.030	61.014–1.04	<.001		1.048	1.025–1.072	<.001		1.030	1.014–1.048	<.001
RE		1.020	1.007–1.033	.003		1.014	71.003–1.02	.011		1.024	1.010–1.038	<.001		1.014	1.004–1.025	.009
MH		1.041	1.015–1.068	.002		1.019	40.999–1.039	.062		1.047	1.019–1.075	<.001		1.020	1.000–1.041	.053
BMI^a^	60				143				60				143			
Obese		1				1				1				1		
Overweight		2.727	0.856–8.685	.090		2.889	1.054–7.91	.039		2.313	0.700–7.641	.169		2.647	0.947–7.394	.063
Normal		4.519	1.414–14.448	.011		4.063	71.443–11.439	.008		4.954	1.516–16.182	.008		4.241	1.498–12.009	.007
BMI, kg/m^2^	61	0.887	0.810–0.972	.010	143	0.931	0.864–1.003	.061	61	0.883	0.804–0.970	.040	143	0.931	0.864–1.002	.057
VFA, cm^2^	61	0.986	0.977–0.994	<.001	142	0.991	0.984–0.998	.009	61	0.987	0.979–0.996	.003	142	0.991	0.984–0.998	.013
Not MVPA^b^	61	1			142	1										
MVPA		1.250	0.456–3.430	.665		0.990	0.400–2.448	.982								
Unhealthy diet^c^	61	1			142	1			61	1						
Healthy		2.020	0.701–5.820	.193		1.216	0.489–3.028	.674		1.702	0.578–5.010	.335				
Unhealthy alcohol intake	60	1				1										
Healthy^d^		1.349	0.517–3.520	.541	142	1.186	0.501–2.810	.698								
No KOA^e^	61	1.250	0.456–3.430	.665	144	1.689	0.670–4.259	.267								

NCP: no chronic pain; CRP: chronic regional pain; CWP: chronic widespread pain; HRQoL: health-related quality of life; PF: physical functioning; RP: role function – physical aspect; BP: bodily pain; GH: general health perception; VT: vitality; SF: social functioning; RE: role function – emotion aspect; MH: mental health; BMI: body mass index; VFA: visceral fat area; MVPA: being moderate and/or vigorous physical active; KOA: radiographic knee osteoarthritis.

Analysed by multinominal logistic regression (CWP as reference, *n* = 27). Variables with a *p* value of ≤.25 were included in the multivariate analysis adjusted for age and sex.

^a^ Normal BMI = 18.5–24.9 kg/m^2^; overweight = 25.0–29.9; obesity ≥30.0.

^b^ WHO recommendations: 150–300 min of moderate-intensity and/or 75–150 min of vigorous-intensity.

^c^ Vegetables and fruit every day, fish 2/week, breakfast most days, pastries a few times/week.

^d^ Healthy intake = ≤4 units/week.

^e^ Having a score <1 on the Ahlbäck scale for knee osteoarthritis.

## Discussion

This study aimed to examine the associations of HRQoL and lifestyle habits with chronic pain at a two-year follow-up in individuals with knee pain. The result showed that individuals with NCP (having only knee pain), reported better HRQoL in all SF-36 sub-scales than those with CRP and CWP at baseline, as did those with CRP compared to CWP. Compared to individuals with no knee pain, those in the NCP group reported worse HRQoL on BP, as expected, but better HRQoL in RP, SF and MH [6]. Significant lower scores in HRQoL were found for all eight sub-scales in individuals with CWP compared to those with CRP at baseline, which aligns with previous research [6,29]. Less spread pain equalled less interference on various aspects of HRQoL. These results are in line with previous research stating not everyone with chronic pain has pain-related interference (i.e. perceived disruption in daily activities, relationships, roles and employment resulting from pain) [9].

Most participants remained in the same pain group as in baseline, but approximately one in eight reported less pain after two years. Overall, few changes in HRQoL and lifestyle habits were found. However, the group that transitioned to less pain reported significantly better GH after two years compared to baseline, suggesting that the overall sense of HRQoL is an important part of pain transitioning early on. Moreover, better HRQoL at baseline was associated with NCP at the two-year follow-up, and on the contrary, worse HRQoL in most sub-scales was associated with CRP and all sub-scales with CWP. Stating that a good HRQoL is important for maintaining NCP. Contrary to the present study’s results, previous research has found psychological factors such as mental distress and emotional exhaustion can predict increased pain after two years [30]. In addition, having low self-efficacy and insufficient social and emotional support have been found to predict chronic pain after 18 months [31]. Better HRQoL was associated with transitioning to less pain from CWP in individuals with rheumatoid arthritis over a seven-year perspective [32]. Another study highlighted the importance of being observant of poorer general health regarding the increase of pain from a one-year perspective [33]. Hence, various concepts of HRQoL may be associated with pain development over a longer period of time in individuals with knee pain.

Moreover, significant improvements were found in the HRQoL sub-scales RP and VT among the individuals with unchanged CWP. Several factors could explain these findings, but it is probably a power issue where the small groups make few individual changes visible.

The prevalence of overweight and obesity was overall high but highest in those reporting CWP. Being overweight, obese and having high-risk VFA at baseline were associated with reporting CWP after two years. On the other hand, the absence of overweight, obesity and high-risk VFA were associated with reporting NCP at the two-year follow-up. These results are in line with previous research stating that chronic pain is more prevalent in individuals who are overweight, ascending with increasing BMI [15,34]. However, over the two years, no significant changes were found in weight or VFA among those transitioning to more or less pain. These results align with another study, which found that BMI did not influence pain transitioning in one year [33]. However, in a longer perspective of seven years, normal BMI was associated with transitioning to less pain from CWP in individuals with rheumatoid arthritis [32]. The high prevalence of overweight and obesity constitutes an obstacle to studying pain transitions over time. Excessive fat tissue releases adipokines, such as leptin, causing a low-level inflammation, which stimulates the pain system and most likely contributes to central sensitisation and the pain’s persistence [13,34]. Increased leptin levels have been found in those with CWP in the HALLOA cohort [35]. To conclude, changes in overweight and excessive VFA were not shown in pain transitioning in individuals with knee pain, but it is possible they have an impact over a more extended time or in a larger sample.

In individuals with NCP, 70% reported meeting the physical activity recommendations, whereas 60% in those with CWP. However, being physically active at baseline was not associated with less pain at the follow-up, and few changed their physical activity over the two years. The groups with unchanged NCP showed a statistically significant change in numbers meeting the recommendations for physical activity, and the opposite was found in those transitioning to more pain, where fewer meet the recommendations. However, these results are not considered clinically relevant.

Moreover, the presence of a healthy diet was overall low in all pain groups at baseline. A healthy diet was not associated with reporting NCP, CRP or CWP at the two-year follow-up, and no changes over time were found in those transitioning to more or less pain. The present study’s results did not cohere with previous studies stating that physical activity and diet have a role in developing CRP and CWP [34,36,37]. On the other hand, being regularly physically active does not necessarily mean having less pain but appreciating the positive benefits of physical abilities (e.g. walking) [37]. Another study found that being physically active every week was positively associated with PF and GH, regardless of pain among individuals with rheumatic and musculoskeletal disease [37].

Regarding diet, it has been observed that individuals with chronic pain have excessive calorie consumption and high intakes of sugar and fat [36]. In addition, foods that contain sugar can temporarily increase the pain threshold and thus have an analgesic effect, which may explain part of the strong association between overweight/obesity and chronic pain [34]. Studies have also found that homeostatic imbalance, which can affect eating behaviours, is altered in individuals with chronic pain, resulting in a greater challenge for choosing a healthy diet [34].

Even though physical activity and diet were not associated with pain development in two years, they can affect other important aspects of an individual’s HRQoL. In addition, physical activity reduces the risk of non-communicable diseases [38], depression and functional immobility [39]. An unhealthy diet can lead to overweight, which is a leading cause of cardiovascular diseases and some cancers [40]. Thus, individuals with knee pain should still be encouraged to exercise and have a healthy diet for other health benefits.

One strength of this study is that all participants had knee pain at baseline, making the sample suitable for the aim, given that individuals with knee pain are more likely to develop CWP [3]. However, a limitation of the study was the already high prevalence of chronic pain other than the knee at baseline. Another limitation is that the diet variable does not include amounts such as calories, and the result should be interpreted accordingly. Most individuals remained at the same pain status as in baseline, indicating that two years is too short to investigate if HRQoL and lifestyle habits impact the pain distribution on a group level in a cohort design. Reflecting on the rapid progression from clinical to radiographic KOA found in the cohort and the associations with HRQoL and overweight [18], studying the possible associations on pain transitions in the same time frame of two years was considered relevant. However, the observational nature of the design makes it difficult to expect that lifestyle habits will change spontaneously. A longer follow-up with a larger sample is needed for further insights into how HRQoL and lifestyle habits influence pain transitions without intervention. Variables other than those investigated in the present study could be significant in transition to and from chronic pain in individuals with knee pain. Future studies should also consider early interventions in individuals with knee pain, targeting overweight/obesity and various aspects of HRQoL to prevent the development of CWP. Lastly, the number of comparisons made in the study may elevate the risk of incorrectly rejecting the null hypothesis, and *p* values should be interpreted accordingly.

## Conclusions

Various concepts of HRQoL and body weight were associated with pain status after two years in individuals with knee pain. However, few individuals moved between the pain groups over two years, and few changed their way of living. Those transitioning to less pain at the two-year follow-up increased their general health significantly, suggesting that the overall HRQoL has a role early on among individuals with knee pain who transition to less pain.

When visiting primary care for knee pain, especially if reporting poorer health or being overweight, individuals should receive broader attention that addresses more than just the knee pain. Future studies with a larger sample and more prolonged duration should investigate the associations further.
